# Book Notices

**Published:** 1897-07

**Authors:** 


### Book Notices.

**The** *American Monthly Review of Reviews* for July contains a variety of important contributed articles. Among these we note Edward Clay's able and interesting character sketch of President Seth Low, Dr. Gould's exposition of the plans of the City and Suburban Homes Company, of New York City, for a model suburban settlement; Baron de Coubertin's vivacious account of "The Revival of the French Universities," General Greely's survey of "Higher Deaf-mute Education in America," and Sylvester Baxter's sympathetic review of Edward Bellamy's new book.

### IN PREPARATION FOR EARLY PUBLICATION.

**An American Text-book of Genito-Urinary and Skin Diseases.**—Edited by L. Bolton Bangs, M. D., Late Professor of Genito-Urinary and Venereal Diseases, New York Post-Graduate Medical School and Hospital, and William A. Hardaway, M. D., Professor of Diseases of the Skin, Missouri Medical College.

**An American Text-book of Diseases of the Eye, Ear, Nose and Throat.**—Edited by G. E. de Schweinitz, M. D., Professor of Ophthalmology in the Jefferson Medical College, and B. Alexander Randall, M. D., Professor of Diseases of the Ear in the University of Pennsylvania and in the Philadelphia Polyclinic.

**Macdonald's Surgical Diagnosis and Treatment.**—Surgical Diagnosis and Treatment. By J. W. Macdonald, M. D., Graduate of Medicine of the University of Edinburgh; Licentiate of the Royal College of Surgeons, Edinburgh; Professor of the Practice of Surgery and of Clinical Surgery, Minneapolis College of Physicians and Surgeons.

**Anders' Theory and Practice of Medicine.**—A Text-book of the Theory and Practice of Medicine. By James M. Anders, M. D., Ph. D., LL. D., Professor of the Theory and Practice of Medicine and of Clinical Medicine, Medico-Chirurgical College, Philadelphia.

**Senn's Genito-Urinary Tuberculosis.**—Tuberculosis of the Genito-Urinary Apparatus, Male and Female. By Nicholas Senn, M. D., Ph. D., LL. D., Professor of the Practice of Surgery and of Clinical Surgery, Rush Medical College, Chicago.

**Penrose's Gynecology.**—A Text-book of Gynecology. By Charles B. Penrose, M. D., Professor of Gynecology, University of Pennsylvania.

**Hirst's Obstetrics.**—A Text-book of Obstetrics. By Barton Cooke Hirst, M. D., Professor of Obstetrics, University of Pennsylvania.

**Moore's Orthopedic Surgery.**—A Manual of Orthopedic Surgery. By James E. Moore, M. D., Professor of Orthopedics and Adjunct Professor of Clinical Surgery, University of Minnesota, College of Medicine and Surgery.

**Heisler's Embryology.**—A Text-book of Embryology. By John C. Heisler, M. D., Prosector to the Professor of Anatomy, Medical Department of the University of Pennsylvania.

**Mallory and Wright's Pathological Technique.**—Pathological Technique. By Frank B. Mallory, A. M., M. D., Assistant Professor of Pathology, Harvard Medical School; Assistant Pathologist to the Boston City Hospital; and James H. Wright, A. M., M. D., Instructor in Pathology, Harvard Medical School; Pathologist to the Massachusetts General Hospital.

**NEW VOLUME IN SANDERS' AID SERIES.**

**Sutton and Giles' Diseases of Women.**—Diseases of Women. By J. Bland Sutton, F. R. C. S., Asst. Surgeon to Middlesex Hospital, and Surgeon to Chelsea Hospital, London; and Arthur E. Giles, M. D., B. Sc. Lond., F. R. C. S. Edin., Asst. Surgeon Chelsea Hospital, London.

